# Inhibition of histone acetyltransferase KAT7 suppresses proliferation and migration of breast cancer cells

**DOI:** 10.3389/fonc.2026.1893745

**Published:** 2026-09-03

**Authors:** Wei Du, Xiaoyu Xiao, Jiaxin Zhang, Zhongxin Shi, Minlan Yang

**Affiliations:** 1 Department of Pathology, The Affiliated Hospital of Beihua University, Jilin, Jilin, China; 2 Department of Pathology, School of Medicine, Yangtze University, Jingzhou, Hubei, China; 3 Department of Neurology, Jilin People’s Hospital, Jilin, Jilin, China

**Keywords:** breast cancer, KAT7, migration, proliferation, subtypes

## Abstract

**Background:**

Lysine acetyltransferase 7 (KAT7), also known as HBO1 or MYST2, is highly expressed in multiple cancers. This study aims to explore the expression patterns of KAT7 in breast cancer and its impact on phenotypes of breast cancer cells.

**Methods:**

The expression of KAT7 and the overall survival of patients were analyzed using the online bioinformatics platform GEPIA2. Immunohistochemistry was applied to detect KAT7 protein expression in breast cancer specimens (94 breast cancer tissues and 12 adjacent normal tissues). KAT7 shRNA plasmids were used to inhibit KAT7 expression in MCF-7 and MDAMB231 cells, and this inhibition was verified by western blot analysis. Cell counting kit-8 (CCK8), colony formation, and transwell migration assays were employed to assess the proliferation and migration of breast cancer cells.

**Results:**

KAT7 was highly expressed in breast cancer and correlated with poor patient prognosis. KAT7 expression was significantly higher in breast cancer than in adjacent non-cancerous tissue. 67 of 94 breast cancer samples tested positive for KAT7. KAT7 was significantly negatively correlated with estrogen receptor (ER), progesterone receptor (PR), and Ki67, and was significantly positively correlated with human epidermal growth factor receptor 2 (HER2). KAT7 expression is strongly associated with tumor subtypes. Inhibition of KAT7 significantly suppressed growth, clonogenic formation, and migration in MCF-7 and MDAMB231 cells.

**Conclusions:**

Our findings demonstrate that KAT7 was highly expressed in breast cancer, and inhibition of KAT7 suppressed proliferation and migration of breast cancer cells.

## 1 Introduction

The KAT7 gene, officially known as lysine acetyltransferase 7, is located on human chromosome 17q21.33. It encodes a protein belonging to the MYST family of lysine acetyltransferases, characterized by a zinc finger C2HC-type domain. KAT7 functions as a key subunit of the histone acetyltransferase, binding to ORC1 (HBO1) multimeric complex and exhibiting histone H4-specific acetyltransferase activity (1). Notably, it catalyzes the transfer of an acetyl group from acetyl-CoA to lysine 14 of histone H3, forming the H3K14ac modification (2). Elevated levels of H3K14ac at gene promoter regions are widely associated with active transcription and enhanced expression of downstream genes. KAT7 plays a pivotal role in various biological processes. Through histone acetylation, it modulates chromatin structure and regulates the expression of specific genes (3). It is also involved in cell cycle control (4, 5), thereby influencing cellular proliferation and growth. Furthermore, KAT7 contributes to DNA damage repair mechanisms through its acetylation activity, helping maintain genomic stability (6). In addition to its enzymatic functions, KAT7 interacts with a wide array of proteins, integrating into diverse regulatory networks that impact both cellular and organismal physiology.

Recent research has shown that KAT7 is highly expressed in many types of tumors, where it often serves as an oncogene. A study conducted immunohistochemical detection of KAT7 in 11 types of primary human tumors and found that KAT7 protein is highly expressed in various cancers such as testis, ovary, breast, stomach/esophagus, and bladder (7). It is widely accepted that KAT7 functions as an oncogene across various tumor types, and its expression is linked to poor prognosis in cancers such as gastric cancer (8, 9), colorectal cancer (10), B-cell acute lymphoblastic leukemia (B-ALL) (11), head and neck squamous cell carcinoma (HNSCC) (12), hepatocellular carcinoma (HCC) (13), non-small cell lung cancer (NSCLC) (14), and bladder cancer (15). Importantly, KAT7 functions as a tumor suppressor in specific situations. For instance, the expression of KAT7 is significantly decreased in clear cell renal cell carcinoma (ccRCC) compared to normal kidney tissue (16). Its levels are notably lower in both ccRCC tissue samples and cell lines, with reduced expression linked to distant metastasis and unfavorable outcomes (17). These results emphasize the intricate and context-dependent role of KAT7 in cancer development, highlighting its tissue-specific roles in various types of cancer.

Breast cancer is a leading cause of both death and new cases among women worldwide. In 2022, there were 2.3 million new breast cancer diagnoses globally, leading to 670,000 deaths. It is the most common cancer in women, representing 25% of all cancer cases and 15.5% of cancer-related fatalities (18). According to the eighth edition of the American Joint Committee on Cancer (AJCC) breast cancer staging guidelines and the 2011 St. Gallen International Expert Consensus on early breast cancer, breast cancer is clinically classified into four molecular subtypes based on the presence of estrogen receptor (ER), progesterone receptor (PR), and human epidermal growth factor receptor 2 (HER2): Luminal A, Luminal B, HER2 positive, and Triple negative. Breast cancer is highly heterogeneous, with its histological subtypes and grading playing key roles in evaluating malignancy and predicting prognosis.

Currently, accurate classification of breast cancer is crucial for predicting patient outcomes and guiding treatment choices. In breast cancer, KAT7 is overexpressed and is linked to poorer survival rates (19). Research has shown that HER2-positive cancers have the highest rate of high-level gene amplification, whereas triple-negative breast cancer exhibits the greatest overall frequency of copy number gains. Additionally, triple-negative breast cancer more commonly shows loss of PTEN and mutations in RB1, which may contribute to genomic instability. Within the amplicon near 45 Mbp on chromosome 17q21, KAT7 has been identified as a potential oncogene and is associated with the ER+/PR+ and HER2+ breast cancer subtypes (20). KAT7 is highly expressed in breast cancer tissues and shows a significant correlation with estrogen receptor α (ERα) and PR. In ERα-positive tumors, KAT7 protein levels are positively associated with histological grade, while no such correlation is observed in ERα-negative tumors (21). KAT7 can influence the expression pattern of ERα by promoting its instability through lysine 48-linked ubiquitination in breast cancer. The acetyltransferase activity of KAT7 is linked to its role in ERα ubiquitination. Additionally, KAT7 regulates ERα-mediated transcription. Both the deletion of KAT7 and treatment with anti-estrogen agents significantly suppress the growth of breast cancer cell lines (22). KAT7’s regulation of ER stability in breast cancer could offer a new approach for treating ER-positive breast cancer. Elevated KAT7 expression also enhances the anchorage-independent growth of breast cancer cells (20). Moreover, in breast cancer, KAT7 facilitates the accumulation of tumor stem cell-like cells by acting as a substrate for cyclin E/CDK2 (23).

Considering the pathological diagnostic patterns of breast cancer, KAT7’s functions, and its role in different breast cancer subtypes, this study mainly investigates the differential expression of KAT7 across distinct breast cancer histological types and the role of KAT7 in various biological phenotypes of breast cancer cell lines, aiming to explore the potential of targeting KAT7 for breast cancer treatment.

## 2 Materials and methods

### 2.1 Expression and survival analysis of KAT7

GEPIA2 is a software tool designed to analyze RNA sequencing expression data from numerous tumor and normal samples collected through the TCGA and GTEx projects (24). Differential KAT7 expression and survival analysis of breast cancer patients were analyzed using GEPIA2 software (http://gepia2.cancer-pku.cn).

### 2.2 Clinical samples

Paraffin-embedded sections of 94 breast cancer tissues and 12 adjacent normal tissues from the Department of Pathology, The Affiliated Hospital of Beihua University, collected between December 2021 and December 2025, were selected for this study. Clinical and pathological data collected included patient age, tumor diameter, tumor location, infiltration, TNM staging, lymph node metastasis, and ER, PR, HER2, and Ki67 expression. This study utilized specimens approved by the Medical Ethics Committee of Beihua University Affiliated Hospital (Approval No. 20250056).

### 2.3 Cell culture, plasmids, and transfection

MDAMB231 and MCF-7 breast cancer cells were obtained from the Cell Bank of Type Culture Collection of the Chinese Academy of Sciences. All cells have undergone STR testing and were free from mycoplasma contamination. 293T cells were received as a gift from Wuhan University. Cells were cultured in complete Dulbecco’s modified Eagle’s medium (DMEM, servicebio, G4523, Wuhan, China) supplemented with 10% fetal bovine serum (FBS, Procell, 164210, Wuhan, China) under 5% CO_2_ at 37°C. A shRNA fragment was inserted into the BamHI and EcoRI sites of HSH169661-LVRU6GP vector (GeneCopoeiaTM, HSH169661, Guangzhou, China) for the production of the shKAT7 lentiviral particles. Three different target sequences were synthesized. Sequence A: 5’-GGATAAGCAGATAGAAGAAAG-3’, Sequence B: 5’-GGAGAAGTTAAGGCTGCAAGG-3’, Sequence C: 5’-GCAACTTTGCATGAGACTATC-3’. HEK293T cells were transfected with either the shRNA or the scramble control vector. The culture medium containing viral particles was harvested 48 h later and was used to infect breast cancer cell lines and then selected with puromycin. PEI MAX (PEI 40K) (Polysciences, 24765, Warrington, PA, USA) was used to transfect shRNA.

### 2.4 Immunohistochemistry staining (IHC)

Paraffin sections were deparaffinized and rehydrated, followed by 15 min antigen retrieval with sodium citrate buffer (Solarbio, C1013, Beijing, China) before cooling to room temperature. Staining was performed using the UltraSensitive™ SP IHC Kit (Maxin Biotechnologies, KIT-9720, Fuzhou, China) according to the manufacturer’s protocol. Add 1/500 diluted KAT7 primary antibody (Proteintech, 13751-1-AP, Wuhan, China) and incubate overnight at 4°C. After staining, dehydrated and mounted. An upright microscope (Leica, DM4B, Wetzlar, Germany) was used to observe and photograph. At least 5 effective fields under 200× magnification were examined for evaluating KAT7 expression levels in breast cancer tissue, using a semi-quantitative integration method. Scoring was based on staining intensity: No color: 0; Pale yellow: 1; Deep yellow: 2; Brownish: 3, and positive cell percentage: ≤25%: 1, 26%–50%: 2; 51%–75%: 3; ≥76%: 4. The staining intensity score and positive cell rate score for each slide were multiplied. A total score of ≥6 was considered positive, while <6 was negative.

### 2.5 Western blot analysis

Cells were lysed on ice in RIPA buffer (Beyotime, P0013B, Shanghai, China). The protein concentration was determined by the BCA protein assay kit (Beyotime, P0010, Shanghai, China). A total of 5 μg of cell lysate was separated by 12% SDS-PAGE, transferred onto a 0.45 μm PVDF membrane (Millipore, IPVH00010, Shanghai, China), and blocked with 5% skimmed milk for 2 h at room temperature. The membrane was then incubated with primary antibodies, 1/1000 diluted KAT7 (Proteintech, 13751-1-AP, Wuhan, China) and 1/2000 diluted β-actin (Proteintech, 20536-1-AP, Wuhan, China) at 4°C overnight. After that, the membranes were incubated with HRP-conjugated secondary antibodies (Proteintech, SA00001-2, Wuhan, China) for 2 h at room temperature and were detected with ECL reagent (Meilunbio, MA0186, Dalian, China). The blots intensity was quantified using ImageJ software (Bio-Rad, CA, USA). ImageJ software was used to analyze the gray value of the blots, and the KAT7 gray value was divided by the β-actin gray value of the same sample for normalization. When performing statistical analysis, Unpaired t-test analysis was performed after shKAT7/shNC processing.

### 2.6 Cell Counting Kit-8 (CCK8) assay

Seed cells at 2×10³ cells/well into a 96-well plate with three replicate wells per group. An equal volume of PBS (0.01 M, pH 7.4) was added to the surrounding wells and incubated in a cell culture incubator. After cells adhered and grew, 10 μL of CCK8 reagent (Beyotime, C0038, Shanghai, China) was added to each well on days 0, 1, 2, 3, 4, 5, and 6. Then incubated at 37°C in a 5% CO_2_ incubator for 1.5 h, the absorbance values were determined by measuring absorbance at 450 nm with a Microplate reader (Molecular Devices, SpectraMax iD3s, Shanghai, China). A cell growth curve was plotted. The experimental procedure refers to a previous study (25).

### 2.7 Colony formation assay

Cells were inoculated in a 6-well plate with 500 cells/well. Cell colonies were fixed with 4% paraformaldehyde for 15 min when they were just about to be visible to the naked eye (no less than 50 cells per clone) and then visualized by 1% crystal violet staining (Beyotime, C0121, Shanghai, China). The experimental procedure refers to a previous study (25).

### 2.8 Transwell migration assay

DMEM medium supplemented with 20% FBS was added to the bottom chambers, and then cells suspended in the DMEM medium were seeded into the top chambers. To eliminate the impact of cell division on migration ability, 1 μg/ml mitomycin C (MCE, HY-13316, Shanghai, China) was used to pretreat for 1 h before plating into the chambers. After incubation for 24 h, noninvaded cells on the upper side of the filter membranes were removed by scraping. Migrated cells on the underside were fixed with 4% paraformaldehyde for 15 min and stained with 1% crystal violet for 15 min at room temperature. Cells on the bottom of the chamber were counted under an inverted phase-contrast microscope (Sunny, IRX50, Ningbo, China). The experimental procedure refers to a previous study (25).

### 2.9 Statistical analysis

SPSS 25.0 software (IBM, Armonk, NY, USA) was applied for statistical analysis. Categorical data were expressed as n or percentage and processed using Chi-square test. Fisher’s exact test was used when the theoretical frequency of samples was smaller than 1; indicators with statistical significance were given multivariate logistic regression analysis. In addition, ER, PR, HER2, Ki67 were correlated with KAT7 using Pearson correlation coefficients. All the *in vitro* experiments were independently performed at least three times, five different fields were captured in Transwell migration assay, with the data presented as the mean ( ± standard error mean), and each time similar results were obtained. A student’s t-test was applied for statistical analysis between two groups, and analyses of variance (one-way ANOVAs) were applied for multiple group comparisons. The statistical analysis was performed using GraphPad Prism 8.0 software (GraphPad, San Diego, CA, USA). *p* < 0.05 was considered statistically significant.

## 3 Results

### 3.1 KAT7 is highly expressed in breast cancer

**Figure 1:**
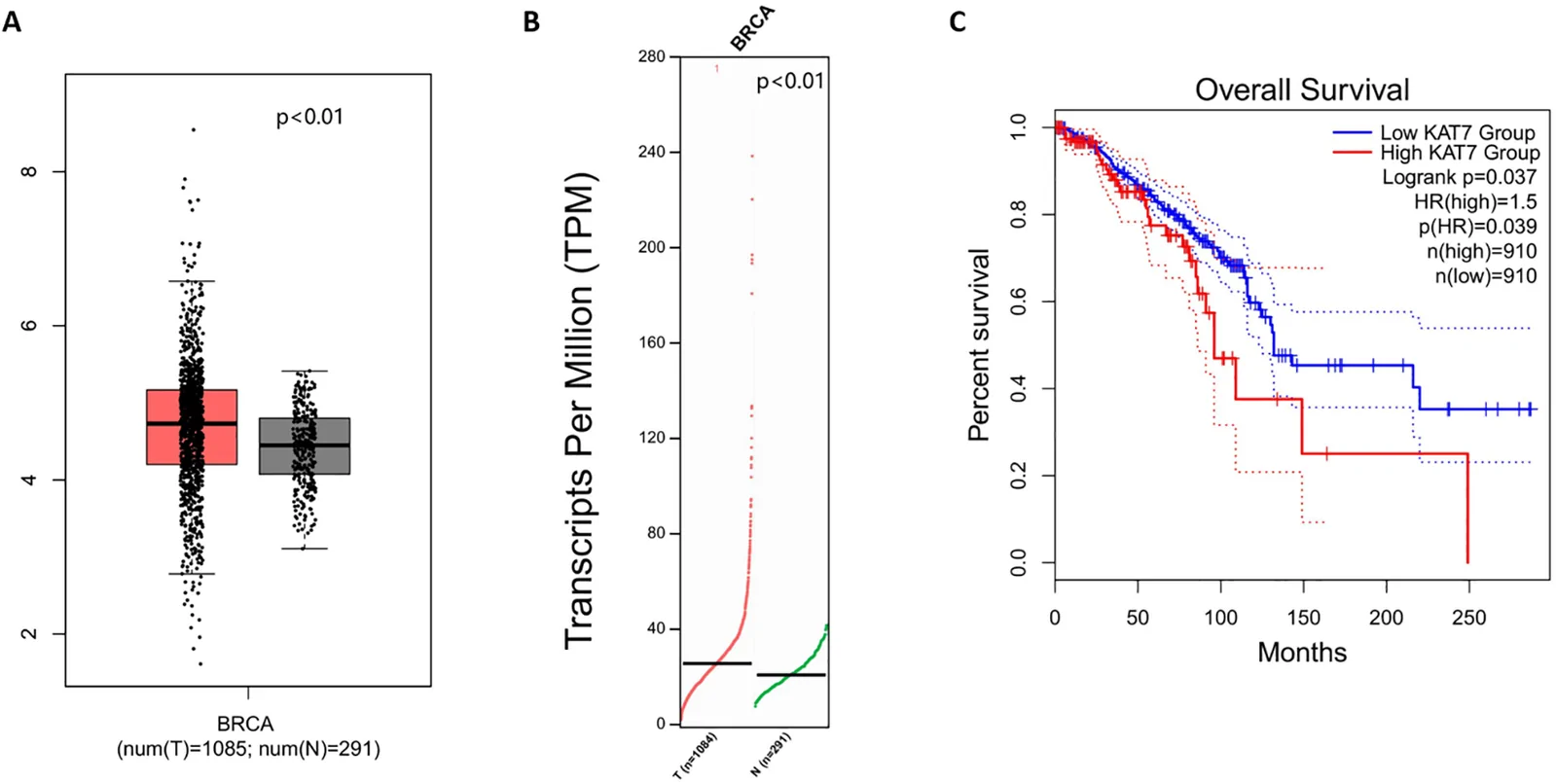
Bioinformatics analysis of KAT7 expression in breast cancer and the correlation between KAT7 expression and patient prognosis. **(A, B)** Analysis of KAT7 expression in breast cancer patients using GEPIA2, *p <*0.01. **(C)** Overall patient survival analysis using GEPIA2, *p <*0.05.

Bioinformatics analysis of KAT7 expression in breast cancer tissues was performed using the GEPIA2 online tool. The results showed that KAT7 expression levels are higher in breast cancer tissues compared to normal tissues (**Figures 1A, B**), based on matched TCGA normal and GTEx data (|log2FC| cutoff = 0.5, *p* cutoff = 0.01). Additionally, overall survival analysis, adjusted with a 95% confidence interval and using cutoff values of 15% for high expression and 85% for low expression, indicated that high KAT7 expression is significantly linked to poorer prognosis (*p* < 0.05) (**Figure 1C**). Taking into account the varying prognoses of different breast cancer subtypes, we utilized GEPIA2 for an online analysis to assess the effect of KAT7 expression on the overall survival of patients with Triple-negative, HER2-positive non-Luminal, Luminal A, and Luminal B breast cancer subtypes. The analysis showed that KAT7 expression was significantly associated only with the prognosis of the Luminal A subtype, where patients exhibiting high KAT7 expression had a poorer prognosis (*p* < 0.05). These findings are presented in **Supplementary Figure 1**.

**Figure 2:**
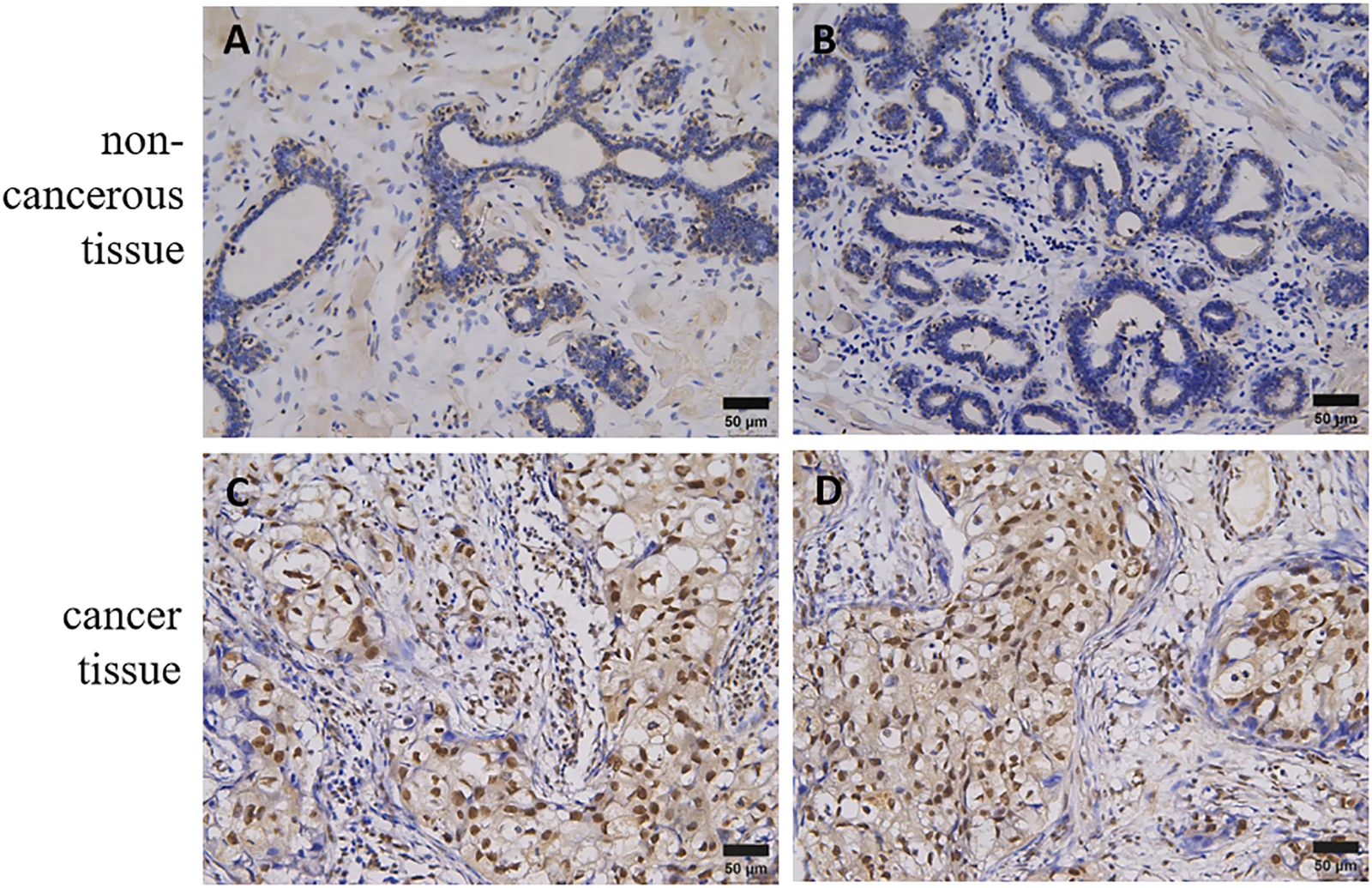
KAT7 is expressed at higher levels in breast cancer than in adjacent non-cancerous tissue. **(A, B)** Adjacent non-cancerous tissue. **(C, D)** Breast cancer tissue. Scale bar 50 μm.

Immunohistochemical staining was conducted on 94 breast cancer tissue samples and 12 adjacent normal tissues. The findings revealed significantly higher KAT7 expression in breast cancer tissues compared to adjacent normal tissues (**Figures 2A–D**). It showed that 67 breast cancer samples (71.277%) tested positive for KAT7 expression, whereas 27 samples (28.723%) were negative. In contrast, among the 12 adjacent normal tissues, only 3 samples (25%) showed positive KAT7 expression, while 9 samples (75%) were negative. This difference was statistically significant, these results are summarized in **Table 1**, demonstrating that KAT7 is markedly overexpressed in human breast cancer tissues.

**Table 1:** Expression of KAT7 in breast cancer and non-cancerous tissues.

Category	Number of cases	KAT7 positive expression (%)	KAT7 negative expression (%)	Chi-square value	*p* value
Breast cancer tissue	94	67(71.277%)	27(28.723%)	10.161	0.001^1^
Non-cancerous tissue	12	3(25.000%)	9(75.000%)		
Total	106	70	36		

^1^, Chi-square test. KAT7, Lysine acetyltransferase 7.

### 3.2 KAT7 expression is associated with the pathological characteristics of breast cancer

Since KAT7 is typically expressed at higher levels in breast cancer, we further analyzed its relationship with clinicopathological characteristics. KAT7 expression did not show a significant association with age, tumor location, tumor size, invasion type, histological grade, lymph node metastasis, or TNM stage. However, KAT7 expression was significantly linked to the presence of ER (*p* < 0.001), PR (*p* < 0.001), HER2 (*p* < 0.001), and Ki67 (*p* < 0.001). Additionally, KAT7 expression was strongly associated with different breast cancer subtypes (*p* < 0.001). These results are summarized in **Table 2**. Notably, in the Luminal A subtype, which has the most favorable prognosis, KAT7 was generally expressed at low levels, whereas in other breast cancer subtypes, KAT7 expression was typically elevated.

**Table 2:** The relationship between expression of KAT7 and clinical-pathological characteristics.

Category	Number of cases	KAT7 positive expression (%)	KAT7 negative expression (%)	Chi-square value	*p* value
Age (years)				0.005	0.945
≤50	17	12(70.588%)	5(29.412%)		
>50	77	55(71.429%)	22(28.571%)		
Grade				2.869	0.238
I	3	2(66.667%)	1(33.333%)		
II	54	35(64.815)	19(35.185%)		
III	37	30(81.081%)	7(18.919%)		
Histopathological type				0.196	0.658
Invasive Carcinoma of No Special Type	89	63(70.787%)	26(29.213%)		
Invasive Carcinoma of Special Type	5	4(80.000%)	1(20.000%)		
Lymph node metastasis				1.16	0.281
yes	21	13(61.905%)	8(38.095%)		
no	73	54(73.973%)	19(26.027%)		
Tumor site				0.051	0.822
Left breast	40	29(72.5%)	11(27.5%)		
Right breast	54	38(70.370%)	16(29.630%)		
ER status				7.545	< 0.01
Positive	64	40(61.905%)	24(38.095%)		
Negative	30	27(90.323%)	3(9.677%)		
PR status				15.319	< 0.001
Positive	54	30(55.556%)	24(44.444%)		
Negative	40	37(92.500%)	3(7.500%)		
HER2 status				12.271	< 0.001
Positive	23	23(100%)	0(0.000)		
Negative	71	44(61.972%)	27(38.028%)		
Ki67 status				22.881	< 0.001
+	17	5(29.412%)	12(70.588%)		
++	29	19(63.517%)	10(34.483%)		
+++	48	43(89.583%)	5(10.417%)		
TNM stage				0.004	0.949
I	39	27(69.231%)	12(30.769%)		
II	47	34(72.340%)	13(27.660%)		
III	8	6(75.000%)	2(25.000%)		
Tumor diameter				0.306	0.580
≤ 2.2cm	48	33(68.750%)	15(31.250%)		
>2.2cm	46	34(73.913%)	12(26.087%)		
Subtypes				67.860	< 0.001
Luminal A	25	2(8.000%)	23(92.000%)		
Luminal B (HER2 positive)	11	11(100%)	0(0.000)	28.025	<0.001^2^
Luminal B (HER2 negative)	26	25(96.154%)	1(3.846%)	39.755	<0.001^2^
HER2 enriched	12	12(100%)	0(0.000)	29.177	<0.001^2^
Triple negative	20	17(85%)	3(15%)	27.005	<0.001^2^

^2^,Chi-square test compared with Luminal A, *p* < 0.001. ER, estrogen receptor; HER2, human epidermal growth factor receptor 2; PR, progesterone receptor.

To better understand the relationship, we conducted a Pearson correlation analysis between these biomarkers and KAT7 expression. Our results showed that KAT7 had a significant negative correlation with ER (r = -0.283; *p* < 0.01), PR (r = -0.404; *p* < 0.01), and Ki67 (r = -0.490; *p* < 0.01). In contrast, KAT7 was significantly positively correlated with HER2 (r = 0.361; *p* < 0.01).These results are summarized in **Table 3**.

**Table 3:** Pearson correlation coefficient Matrix between KAT7 and breast cancer biomarkers.

Variables	ER	PR	HER2	Ki67	KAT7
ER	1				
PR	0.795**	1			
HER2	-0.141	-0.211*	1		
Ki67	0.242*	0.276**	-0.208*	1	
KAT7	-0.283**	-0.404**	0.361**	-0.490**	1

Pearson correlation coefficient analysis. The value is the Pearson correlation coefficient. *p < 0.05; **p < 0.01.

A multivariate logistic regression analysis was conducted to assess the relationships between various clinical and pathological factors and KAT7 expression. The findings indicated that only the breast cancer subtype was significantly linked to KAT7 expression (*p* < 0.01). Specifically, the regression coefficient for subtype was *B* = −3.019, which corresponds to an odds ratio (OR) of 0.049 (95% confidence interval: 0.009 ~0.270). In contrast, none of the other variables analyzed—including histopathological type, age, histologic grade, tumor location, ER status, PR status, HER2 status, Ki67, lymph node metastasis, TNM stage, and tumor size—demonstrated statistically significant associations (as presented in **Table 4**).

**Table 4:** Multivariate logistic regression analysis of KAT7 expression and clinical-pathological characteristics in breast cancer patients.

Variables	*B*	*p* value	Exp(*B*)	95% CI
Histopathological type	-0.874	0.822	0.417	0~844.171
Age	-0.631	0.614	0.532	0.046~6.171
Grade	1.723	0.229	5.601	0.338~92.682
Tumor site	-0.639	0.532	0.528	0.071~3.922
ER	24.592	0.998	47872060667.832	0
PR	-17.912	0.999	0	0
HER2	21.683	0.998	2610184822	0
Ki67 status	-0.574	0.58	0.563	0.073~4.314
TNM stage	0.484	0.728	1.623	0.106~24.878
Tumor diameter	-0.016	0.99	0.984	0.077~12.559
Subtypes	-3.019	0.001^3^	0.049	0.009~0.27

^3^, Multivariate logistic regression analysis. *B*, regression coefficient beta; Exp(*B*), exponentiated beta, means odd ratio; 95% CI, 95% confidence interval.

### 3.3 Inhibiting KAT7 expression suppresses breast cancer cell proliferation and migration

**Figure 3:**
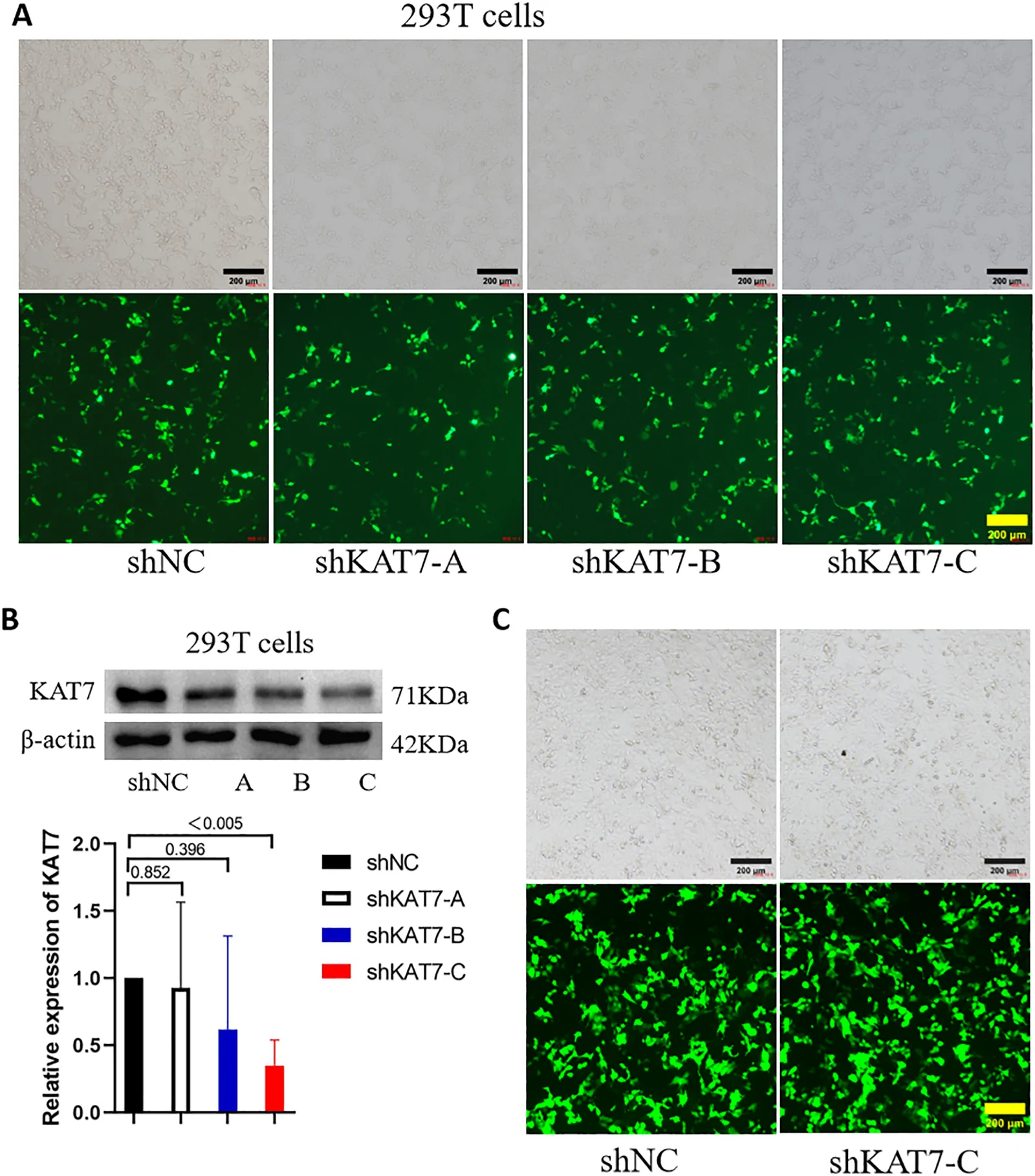
293T cells transfected with KAT7 shRNA to screen for the optimal sequence. **(A)** Fluorescence image of transient transfection. Scale bar 200 μm. **(B)** Western blot assay assessment of inhibition efficiency. **(C)** Fluorescence image of 293T cells packaging shKAT7 lentivirus particles. Scale bar 200 μm.

To determine whether KAT7 functions as an oncogene in breast cancer, given its overexpression in breast tumors, we employed shRNA to suppress KAT7 expression and validate its role in the malignant phenotypes of breast cancer cells. First, through transient transfection of 293T cells, we screened for the optimal interference sequence and found that sequence C exhibited the most significant suppression efficiency (*p <*0.005) (**Figures 3A–C**). Therefore, we packaged the sh-KAT7-C plasmid into lentiviral vectors and infected breast cancer cell lines with the collected viral supernatants.

**Figure 4:**
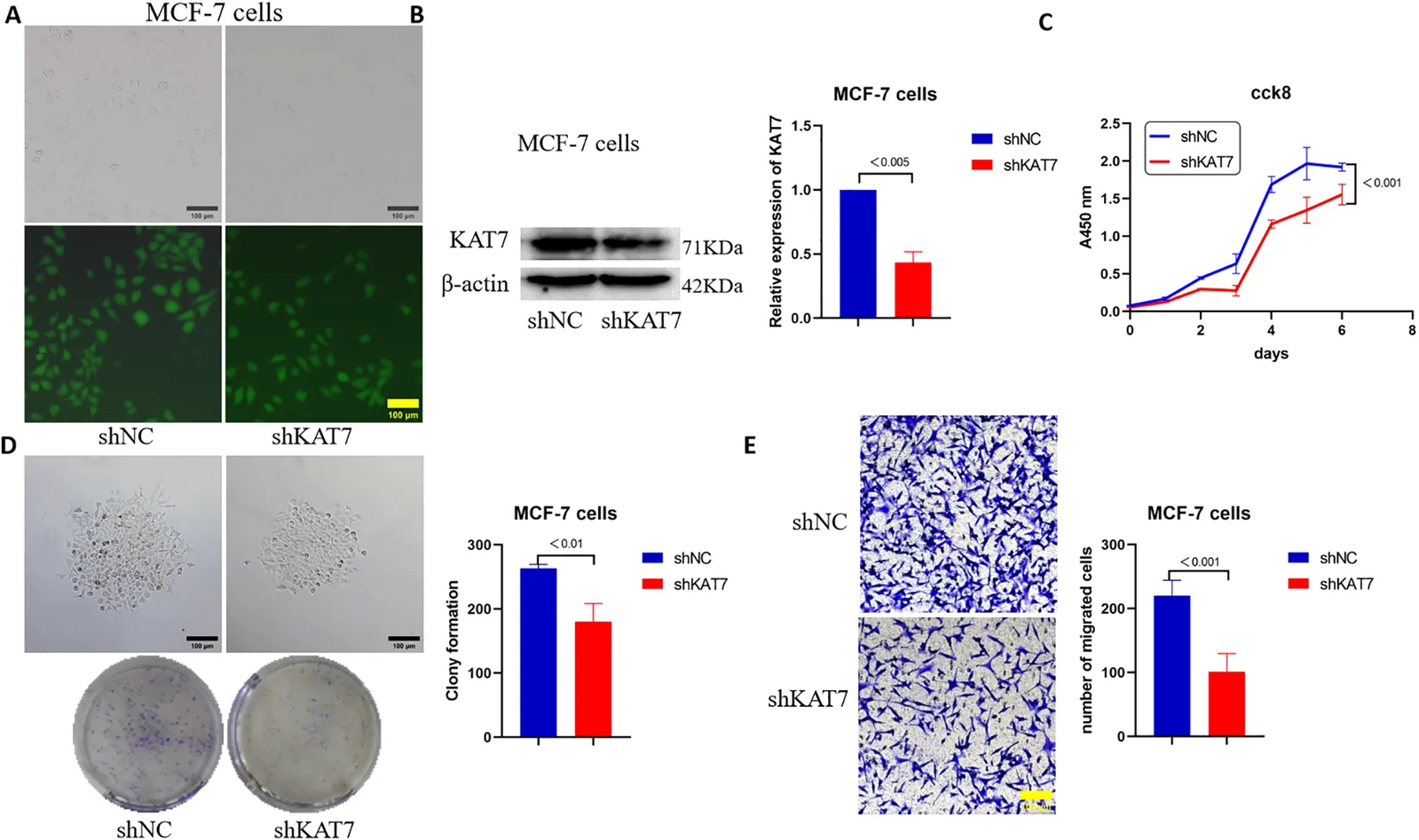
Inhibition of KAT7 suppresses MCF-7 cell proliferation, colony formation, and migration. **(A)** Fluorescence image of MCF-7 cells infected with the virus. Scale bar 100 μm. **(B)** Western blot assay verified the decreased expression of KAT7. **(C)** The CCK8 assay was used to detect cell proliferation. Scale bar 100 μm. **(D)** The colony formation assay was used to detect cell proliferation. **(E)** The transwell migration assay was used to detect the migration ability of cells. Scale bar 100 μm.

Suppression of KAT7 expression levels in the breast cancer cell line MCF-7 was verified by Western blot analysis (*p* < 0.05) (**Figures 4A, B**). The CCK8 assay assessed the impact of KAT7 knockdown on MCF-7 cell growth, revealing that inhibition of KAT7 significantly suppressed MCF-7 cell proliferation (*p* < 0.001) (**Figure 4C**). Clonogenic assays demonstrated that inhibiting KAT7 significantly suppressed MCF-7 cell clonogenic capacity (*p* < 0.01) (**Figure 4D**). Transwell migration assays revealed that inhibiting KAT7 markedly suppressed MCF-7 cell migration (*p* < 0.001) (**Figure 4E**).

**Figure 5:**
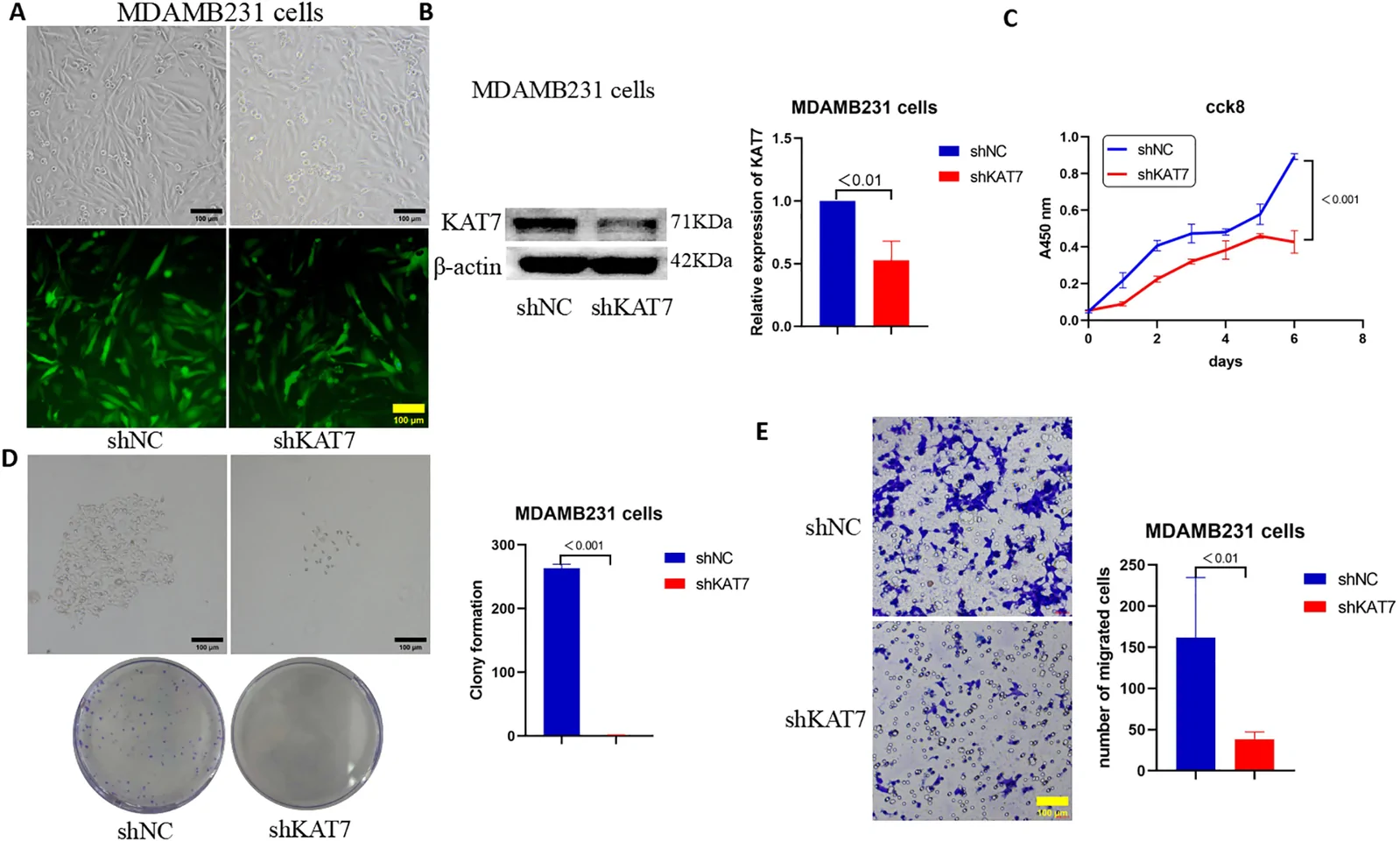
Inhibition of KAT7 suppresses MDAMB231 cell proliferation, colony formation, and migration. **(A)** Fluorescence image of MDAMB231 cells infected with the virus. Scale bar 100 μm. **(B)** Western blot assay verified the decreased expression of KAT7. **(C)** The CCK8 assay was used to detect cell proliferation. **(D)** The colony formation assay was used to detect cell proliferation. Scale bar 100 μm. **(E)** The transwell migration assay was used to detect the migration ability of cells. Scale bar 100 μm.

Due to its distinct biological behavior and clinical pathological characteristics, triple-negative breast cancer carries a poorer prognosis than other subtypes. Therefore, we employed triple-negative breast cancer cell lines for further validation. Lentiviral transduction suppressed KAT7 expression levels in MDAMB231 breast cancer cells, as verified by Western blot analysis (**Figures 5A, B**). The CCK8 assay assessed the impact of KAT7 knockdown on MDAMB231 cell growth, revealing that inhibiting KAT7 significantly suppressed cell proliferation (*p* < 0.001) (**Figure 5C**). A clonogenic assay examined the effect of KAT7 knockdown on MDAMB231 cell growth, revealing that inhibiting KAT7 significantly suppressed the clonogenic capacity of MDAMB231 cells (**Figure 5D**). The transwell migration assay assessed the impact of KAT7 knockdown on MDAMB231 cell migration, demonstrating that inhibiting KAT7 significantly suppressed MDAMB231 cell migration (**Figure 5E**). The same results were achieved in another triple-negative breast cancer cell line Hs578t. Two mixed KAT7 shRNA were used to inhibit KAT7 expression, which was verified by Western blot analysis. We found that inhibition KAT7 suppressed the growth, colony formation, and migration of Hs578t cells (**Supplementary Figures 2A–D**).

## 4 Discussion

In 2022, the three most prevalent cancer types worldwide were lung cancer, breast cancer, and colorectal cancer. Among all malignant tumors in women, breast cancer ranked first in incidence (26). In China alone, approximately 300,000 new cases of breast cancer are diagnosed annually, with a notable trend toward younger age at onset (27). A range of factors contribute to increased breast cancer risk, including advanced age, obesity, harmful alcohol consumption, family history of breast cancer, prior radiation exposure, reproductive history, tobacco use, and postmenopausal hormone therapy (18). Currently, surgery and chemotherapy remain the cornerstone treatments in clinical practice. Clinical management typically relies on imaging, histopathological examination, and tumor marker assessment for diagnosis and prognosis evaluation. Despite their widespread use, these methods have significant limitations, including insufficient specificity and variable applicability, which constrain their effectiveness in accurately predicting disease progression and patient outcomes.

Acetylation exerts diverse effects at both the protein and metabolome levels, participating in a wide range of metabolic processes. Lysine acetyltransferases, often referred to as histone acetyltransferases (HATs), are primarily categorized into four families based on sequence and structural homology: the GNAT/PCAF (GCN5) family, the p300/CBP family, the MYST family, and the SRC/ACTR family. The MYST family consists of five members—KAT5, KAT6A, KAT6B, KAT7, and KAT8—each harboring a highly conserved “MYST” domain that confers acetyltransferase activity. The MYST family plays essential roles in multiple cellular processes, including the regulation of gene transcription, DNA repair, and DNA replication (28). This study revealed that KAT7 expression levels are markedly elevated in breast cancer compared to normal tissues. This finding was consistent with conclusions reported in previous studies. Then we analyzed the correlation between KAT7 expression and the clinicopathological features of breast cancer patients. The status of ER is a key factor in classifying breast cancer, directly influencing treatment decisions and prognosis evaluation. Previous studies have reported that KAT7 acts as a potential oncogene in ER+/PR+ and HER2+ subtypes of breast cancer (20). It is highly expressed in breast cancer and is significantly positively correlated with ERα and PR (21). However, in our study, KAT7 was significantly negatively correlated with ER, PR, and Ki67, which is inconsistent with previous reports. Interesting, it was reported that KAT7 promotes the instability of ERα in breast cancer through lysine 48-linked ubiquitination, thereby inhibiting breast cancer growth by targeting KAT7 (22), which is partly consistent with our results, as we found that KAT7 was significantly negatively correlated with ER, which may be due to KAT7 affecting ER instability. However, there is a lack of relevant research evidence in our current study. Additionally, KAT7 is positively correlated with HER2 in our experiment. Overexpression of HER2 suggests a higher risk of recurrence and poor prognosis in breast cancer (29). Therefore, we speculate that high expression of KAT7 maybe associated with increased risk and poor prognosis in breast cancer. Since our clinical sample lacks risk prediction analysis and prognostic data, our speculation needs to be confirmed by further studies. KAT7 expression was strongly associated with breast cancer subtypes, therefore, we speculate that KAT7 expression may be used as a basis for judging molecular subtypes of breast cancer. As Luminal A subtype has the most favorable prognosis (30), it showed negative expression of KAT7 in Luminal A subtype, suggesting that low levels of KAT7 expression may indicate a more favorable tumor prognosis. As the percentage of Ki67 expression can be used to determine the proportion of cells in the proliferation cycle and is an important indicator in clinical evaluation of tumor proliferation (31), we found that KAT7 was negatively correlated with Ki67 expression, but in the multivariable logistic regression analysis of breast cancer biomarkers, molecular subtypes is still statistically independent factor of KAT7, indicating that KAT7 expression may be used as a basis for classifying molecular subtypes of breast cancer, Of course, a larger sample size of multicenter patients is needed to verify this possibility. *In vitro* experiments were conducted to examine the effect of KAT7 expression levels on breast cancer cells’ growth and colony formation to clarify the relationship between KAT7 and tumor proliferation. Additionally, high Ki67 expression is associated with an increased risk of breast cancer metastasis (32), so we further validated the relationship between KAT7 expression levels and breast cancer cells’ metastatic potential through the transwell migration assay. Inhibition of the expression of KAT7 suppressed the growth, colony formation and migration of MCF-7 and MDAMB231 cells. It is speculated that KAT7 inhibitors may be effective in treating breast cancer, which needs to be verified by clinical studies.

As HER2-positive breast cancer is more aggressive and has a poorer prognosis. Anti-HER2 targeted therapy significantly improves the outcomes for these patients. In our study, KAT7 expression was positively correlated with HER2, suggesting that anti-HER2 targeted therapy may also be effective in KAT7-positive breast cancer. However, the clinical samples in this study lack clinical data related to patient treatment methods and prognosis, so the accuracy of this speculation cannot be confirmed. Additionally, KAT7 promotes the enrichment of cancer stem cells (CSCs) in breast cancer (23), and the plasticity of these stem cells also depends on KAT7 (33). CSCs are a key factor in tumor progression and contribute to the difficulty of eradicating cancer (34). Triple-negative breast cancer is enriched with CSCs and exhibits unique molecular mechanisms and tumor microenvironments that maintain its CSCs phenotype. In our study, inhibition of KAT7 inhibited the proliferation and migration of MCF-7 cells and two triple-negative breast cancer cell lines. Although we found the inhibitory effect of inhibiting KAT7 on tumor proliferation and migration in breast cancer cells, these cell lines cannot verify the therapeutic effect of targeting KAT7 on different molecular subtypes of breast cancer.

Although no KAT7 inhibitors have yet entered clinical trials, KAT6A and its paralog KAT6B (collectively known as KAT6) belong to the MYST family of histone acetyltransferases, which share similar structures and functions with KAT7. These enzymes catalyze the acetylation of histone H3 lysine 23 (H3K23ac) and play a crucial role in activating gene transcription, providing valuable insights for the development of KAT7 inhibitors. Clinical studies have demonstrated that KAT6 inhibitors are significantly effective in treating tumors, including breast cancer. KAT6 regulates multiple cellular processes by acetylating various lysine residues on histone H3 (35). Notably, KAT6A is overexpressed in several solid tumors and promotes ERα protein expression by regulating estrogen receptor 1 (ESR1) transcription. Targeted inhibition of KAT6 suppresses ESR1 transcription, thereby reducing ERα protein levels. Consequently, targeting KAT6 represents a promising therapeutic strategy for estrogen receptor-positive breast cancer (36). Clinical evidence also confirms that KAT6 inhibitors play a significant role in preventing breast cancer metastasis. These inhibitors exhibit durable antitumor activity and favorable safety profiles in ER+/HER2- metastatic breast cancer patients who have undergone multiple prior therapies, identifying the catalytic domains of KAT6A/KAT6B as viable therapeutic targets for malignant tumors and offering a novel treatment approach for metastatic breast cancer (37). Furthermore, KAT6A and KAT7 are associated with NUP98 fusion oncoproteins within chromatin and condensates, jointly driving leukemogenesis. Inhibitors targeting KAT6A and KAT7 can suppress their histone acetyltransferase activity and represent an effective therapeutic strategy for pediatric acute myeloid leukemia (AML) (38). Studies have shown that combining KAT6 inhibitors with KAT7-targeted therapies significantly enhances treatment efficacy against AML (39), providing a rationale for the antitumor potential of KAT7 inhibitors. Currently, several KAT7 inhibitors have been developed; for example, WM-3835 is a cell-permeable, highly potent first-in-class KAT7 inhibitor. WM-3835 markedly suppresses the viability, proliferation, and cell cycle progression of primary human prostate cancer cells, as well as *in vitro* cell migration. In addition to inhibiting H3-H4 acetylation, WM-3835 downregulates the expression of multiple oncogenes in primary castration-resistant prostate cancer cells (40). With ongoing research elucidating the molecular mechanisms by which KAT7 regulates breast cancer, KAT7 inhibitors are expected to be further developed and applied in clinical breast cancer treatment.

This article has several limitations. For instance, although the study involved 94 samples, the sample size was relatively small, and the patients were primarily from Jilin City, Jilin Province, China, which introduces certain regional biases. Moreover, the cases lack information on patient prognosis, which is a major limitation for thoroughly understanding the diagnostic and prognostic roles of KAT7. Additionally, our study did not find any correlation between KAT7 expression and clinicopathological features such as age, tumor location, tumor size, invasion type, histological grade, lymph node metastasis, or TNM stage, which contrasts with previous research. This discrepancy may be attributed to the significant difference in sample sizes between the studies. Therefore, large-scale, multicenter studies remain essential for accurately assessing the role of KAT7 in breast cancer diagnosis and prognosis. Currently, there are conflicting findings regarding the relationship between KAT7 and ER expression patterns in breast cancer and their association. Further research is needed to determine if other mechanisms influence the interaction between these factors. In particular, this study used shRNA to inhibit KAT7 expression, and we are concerned about potential off-target effects. Although in supplementary experiments we used mixed shRNA to conduct research and obtained conclusions consistent with previous experiments, there is still the risk of potential off-target effects. Unfortunately, due to the tight time and the limited research funding available at this stage, we are currently unable to complete experimental verification of multi-target function and response. Additionally, this study used only three breast cancer cell lines and did not include any HER2-positive cell lines. Our results do not prove the mechanism of KAT7 in different molecular subtypes of breast cancer. In addition, the specific molecular mechanisms by which KAT7 regulates the expression of PR, HER2, and Ki67 are not yet understood, representing a promising avenue for future investigation.

## 5 Conclusions

Our data demonstrate that KAT7 is highly expressed in breast cancer and correlates with poor patient prognosis. Its expression is significantly elevated in breast tumor tissues compared to adjacent non-cancerous tissues. KAT7 is significantly negatively correlated with ER, PR, and Ki67, and is significantly positively correlated with HER2 in breast cancer. Inhibiting KAT7 suppresses the malignant biological behaviors of breast cancer cells.

## Data Availability

The datasets presented in this study can be found in online repositories. The names of the repository/repositories and accession number(s) can be found below: In the article.

## Supplementary material

Supplementary Figure 1 Effect of KAT7 expression on Overall Survival of different subtypes of breast cancer.

Supplementary Figure 2 Inhibition of KAT7 suppresses triple-negative breast cancer cell line Hs578t proliferation, colony formation, and migration. **(A)** Western blot assay verified the decreased expression of KAT7. **(B)** The CCK8 assay was used to detect cell proliferation. **(C)** The colony formation assay was used to detect cell proliferation. Scale bar 100 μm. **(D)** The transwell migration assay was used to detect the migration ability of cells. Scale bar 100 μm.
