# Supplementary material for: Inhibition of histone acetyltransferase KAT7 suppresses proliferation and migration of breast cancer cells
Source: Front Oncol. 2026 Sep 3;16:1893745. doi: 10.3389/fonc.2026.1893745 (PMC13583433; doi:10.3389/fonc.2026.1893745)
Supplement: Supplementary file 1 [file SupplementaryFile1.docx]

**SUPPLEMENTARY**

**Supplementary materials and methods**

Hs578t cells were were purchased from Wuhan Procell Biotechnology. Cells were cultured in complete Dulbecco’s modified Eagle’s medium (DMEM, servicebio, G4523, Wuhan, China) supplemented with 10% fetal bovine serum (FBS, Procell, 164210, Wuhan, China), 10μg/ml insulin (Procell, 180432, Wuhan, China) and 1% penicillin/streptomycin under 5% CO_2_ at 37°C.
